# Therapeutic Strategies against Inflammation-Related Diseases: Molecular Mechanisms and Clinical Applications

**DOI:** 10.1155/2015/382730

**Published:** 2015-08-04

**Authors:** Wen-Bin Wu, Wei-Chien Huang

**Affiliations:** ^1^School of Medicine, Fu Jen Catholic University, New Taipei City 24205, Taiwan; ^2^Graduate Institute of Cancer Biology, China Medical University, Taichung 40402, Taiwan

Inflammation is a complicated cellular and molecular process of vascular tissues response to harmful stimuli. It is a protective mechanism by the host to remove the harmful stimuli, initiating the healing process to promote tissue recovery. However, inadequate or chronic inflammation can lead to a lot of diseases, such as atherosclerosis, cardiac valvular disease, cancer, respiratory diseases, and rheumatoid arthritis.

In this special issue, several studies regarding the therapeutic strategies against inflammation-related diseases are published. First of all, interleukin-27, a recently characterized cytokine, was reported to protect cardiomyocyte-like cells against* in vitro* metabolic syndrome. Two articles report the therapeutic potential of Chinese herbal medicine against inflammation-related diseases: Huangqin Tang can ameliorate colitis by regulating effector and regulatory CD4^+^ T cells; the fiber hydrophobic extract of an annual plant of temperate climate zone, Flax (*Linum usitatissimum* L.), can inhibit human skin cell inflammation and enhance wound closure activation* in vitro*. In addition, a review article summarized the characteristics and roles of thrombin, a key inflammation-related serine protease, in osteoarthritic pathogenesis and treatment. We also collected a paper reporting the clinical prognosis relevance of serum cytokines in pancreatic cancer patients.

In summary, knowledge and understanding of these conditions would be helpful to the development of some therapies, which may provide better care to these patients.

